# External Entropy Production and Human Evolution Toward Multi-Body Life

**DOI:** 10.3390/e28060621

**Published:** 2026-06-01

**Authors:** Yasuji Sawada, Kenji Toma

**Affiliations:** 1 Division for Interdisciplinary Advanced Research and Education, Tohoku University, Sendai 980-8578, Japan; toma@fris.tohoku.ac.jp; 2 Frontier Research Institute for Interdisciplinary Sciences, Tohoku University, Sendai 980-8578, Japan; 3 Astronomical Institute, Graduate School of Science, Tohoku University, Sendai 980-8578, Japan

**Keywords:** external entropy production, awareness, non-equilibrium thermodynamics, maximum entropy production principle, evolution toward multi-body life

## Abstract

Ancient human beings started “external entropy production” in a late stage of evolution, in addition to the internal entropy production by which energy was dissipated within the body of life, as previously described consistently with the birth of life by maximum entropy production principle. In this paper, the mechanism for development of external entropy production, which is strongly related with the use of tools and controlling fire, is theoretically investigated. Archaeological data show that the brain size of ancient human beings started to rapidly increase around 2.5 million years ago when the usage of tools and control of fire started. It may be natural to assume that the rapid growth of brain size is related to the growth of awareness that helped cooperation with the other human beings for control of fire. Coupled equations for the growth rate of the brain, including awareness, and for the growth rate of the size of the interacting human beings are analyzed. External entropy production per human being, which is directly related to the group size of cooperating human beings, is estimated to increase on a timescale of ∼20 million years from the beginning after the critical time. This evolution created the coexistence of the internal entropy production of traditional multi-cellular life and the new external entropy production of multi-body life. A psychological problem due to the coexistence of two kinds of entropy production mechanisms in human beings and the concept of technologies based on the present thermodynamic evolution theory are discussed. It is suggested that the evolutionary understanding of the origin of global warming based on external entropy production may be important to create an useful countermeasure.

## 1. Introduction

The essential role of the maximum entropy production principle (MEPP) for the birth and evolution of life was discussed in the review paper [1], in which a new concept of “external entropy production” was introduced in relation to the later evolution of life, and preliminary discussion was made for the present global warming problem caused by the external entropy production of Homo sapiens. The definition of external entropy production is the entropy produced outside the body of life, contrasted with the internal entropy production, which is entropy produced within the body of the life (the word “external entropy production” is found in [2], but with a different meaning). The importance of this concept is clear because the quantity of external entropy production of Homo sapiens is more than two thousand times greater than their internal entropy production and is still increasing rapidly at present.

Thermodynamically, nature selected the evolution of the brain to initiate and increase the function for the external entropy production, as long as the thermodynamic condition for an open system far from equilibrium (FFE) is satisfied. The largest energy dissipation in the modern world comes from mass production, transportation, and domestic air conditioning. The latter started first and was followed by the former two later. In this paper, the mechanism of birth of external entropy production is investigated together with the thermodynamic evolution theory based on the MEPP.

The role of MEPP for birth and evolution was discussed by many researchers, including the present authors [3]. The relation of this principle to the second law of thermodynamics was confirmed by statistical mechanics. The example for this proof is given in [4]. This paper has shown Pt(σ)/Pt(−σ)∼exp(σt) from the second law of thermodynamics, where σ=Q/Tt is the entropy production, *t* is the interval of time over which the system exchanges the heat *Q*, and Pt(σ) is a time-dependent probability distribution in steady states. This equation implies that the probability ratio of an event producing entropy production σ to the probability of an event producing entropy production −σ is greater than unity and increases with σ. This is equivalent to MEPP when there are several choices of events. This discussion is a natural derivation from the second law of thermodynamics and supports the MEPP for the system FFE. This principle was experimentally verified in material science [5,6,7].

## 2. The Growth of Personal Brain and Size of Society

### 2.1. Birth of Signaling Cells and Neurons Supporting Multicellular Life

The relationship between the nervous system and multi-cell bio-system during evolution is not well known. The evolutionary origin of the nervous system has been a matter of long-standing debate [8]. Although it is not clear at present how neuronal systems started functioning among primitive cell systems, it seems more legitimate to ask why cells needed mutual communication. As demonstrated in the review paper [1], the history of evolution is not necessarily the continuation of thermodynamic condition FFE. The continued survival of life depended on the capability to adapt the hard situation. Some examples of adaptation of life to the change of environment were described in the preceding paper [1]. They could manage to decrease entropy production for survival, corresponding to minimum entropy production [9].

A related behavior is found in dictyostelium discoideum (D. D.) [10]. One of the cells among a group of independent cells starts emitting an oscillating chemical signal of cyclic AMP, to attract other cells to join itself and form a temporal multi-cellular system, which subsequently differentiates into prestalk cells and prespore cells. Then, the prestalk cells gather around the whole collection of cells and finally change into a stalk, while the prespore cells climb up the stalk and change into spores at the top of the stalk until the environmental conditions recover. They stay as a collection of single cells when the environmental condition is favored and they try to form a multiple cell system when the environment is unfavored. Also, this change is caused by the transmission of chemical signals among cells. The assumption that intercellular communication helped the survival of monocellular society may be reasonable. This is again related to the failure of the thermodynamic condition FFE. The behavior of D. D. when the thermodynamic condition changed again into a favorite condition implies the general tendency of evolution corresponding to the MEPP.

### 2.2. Development of Brain and Awareness

Although the signal exchange system was helpful in the unfavored condition, it would have finally helped the activity of the multi-cellular system and increased entropy production compared with a uni-cellular system without signal exchange because this signal developed cooperation between cells. In primitive multi-cellular structures such as hydra [11], the motion of tentacles is connected with a ring-like neural network around the mouth. The neural network developed with evolution. In the late stages of evolution, a concentrated neural system called the brain was enhanced. Simultaneously, sensors such as eyes were formed; the information of the sensors is sent to the brain, and the information is processed for the use of the biosystem. The progress of the brain function made modeling possible for human beings and enabled them to predict the outside world. Tools thus invented are essential as the most specified characteristics for distinguishing human beings from other animals. Tools have been developed from stone tools to advanced information carriers and artificial intelligence.

Fire is a result of a chemical change from fuel materials with rich energy to materials of lower energy by a sort of chain reaction [12]. The use of fire, the basis of modern industries, involves controlling a chain reaction and is directly related to external entropy production. It is natural to imagine that tools that the ancient people had used for a long time had helped them to control the chain reaction of fire burning. It is only recently, however, that the technology has developed for creating gigantic products for a variety of human uses together with the technology of a large amount of energy consumption needed for it.

## 3. Critical Number of Neurons for Self-Awareness Necessary for Invention of Tools and Control of Fire

Awareness or self-awareness has been a philosophical and psychological subject for a long time; recently, scientific research of these subjects began due to the development of brain research and computer science [13,14,15,16]. It is imagined that self-awareness was necessary for the invention of tools about 3.3 million years ago [17], as well as control of fire about 1.7–2.0 million years ago [18].

**Figure 1:**
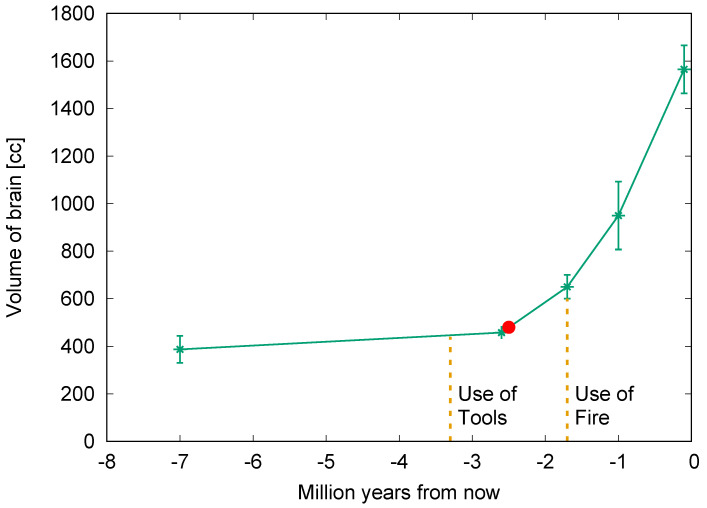
Archaeological data of brain size from chimpanzee to ancient human beings versus time. The green crosses correspond to the different types of homo with chimpanzee. The vertical dashed lines indicate the rough periods when the use of tools and control of fire started. The red point at $T_{c}=-2.5\;\mathrm{million}\;\mathrm{years},N_{c}=480\;$cc is assumed as the critical point for the transition to a phase of new life with in a society and external entropy production.

The appearance times and brain sizes of ancient human beings after chimpanzees are summarized in Table 1. The data of brain size from references [19,20,21,22,23] are plotted with the time of appearance in Figure 1, which clearly shows that brain size started increasing rapidly at a critical point $T_{c}=2.5$ million years ago when the brain was about $N_{c}=480\;$cc. This value may correspond to the time when human beings established self-awareness because it corresponds to the period when the usage of tools and controlling of fire started. Furthermore, this volume of brain corresponds roughly to the brain size of a modern human child at 1.5 years old, when it is believed that they obtain self-awareness [24].

Brain is a complicated network of neurons with synapses and its modeling mechanism of the external world is a vast target of present researcher of brain science [23]. Here, we discuss brain size as the simplest measure of brain function of ancient human beings. It is generally accepted that prefrontal cortex is responsible for higher functions of human being. We used total brain size *N* instead of the volume of prefrontal cortex $N_{p}$ because the information of *N* is archaeologically available, but not of $N_{p}$ for ancient human beings. Therefore, when we assume that $N_{c}$ is 480cc, it does not mean that the critical volume of $N_{p}$ for the birth of awareness is 480cc. It only states that the critical volume of $N_{p}$ should correspond to a value for which *N* is 480cc. The relation between $N_{p}$ and *N* for the present young brains was obtained [25,26], which should be useful for future construction of the model of the birth of awareness for the human child. The legitimacy of using the same relation for ancient human beings and for growing young brains is not clear at present.

By observing Figure 1, together with the consideration in the previous section, it may be reasonable to make the following model for the birth of external entropy production for $N>N_{c}$:

(i) Ancient human beings, around 2.5 million years ago, started to cooperate with each other in an attempt to fabricate tools and control fire, mainly to increase the chance of meeting each other; this was possible due to the gradual increase of the population.

(ii) This cooperation with regard to unaccustomed materials and phenomena accelerated the awareness capacity of the human brain.

(iii) The accelerated brain helped the growth of the external world, represented by the population of a group.

(iv) External entropy production was born as a result of the interaction between the internal world and the external world through awareness.

(v) An increase in the population that uses fire means an increase in external entropy production.

**Figure 2:**
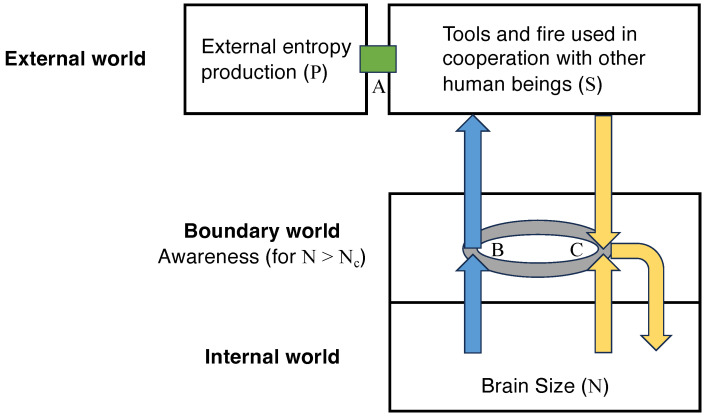
Interaction of internal and external worlds through awareness for $N>N_{c}$. The two worlds interact through the boundary world representing awareness of human beings shown by the gray ring. The yellow arrows represent interaction of the external world and the brain through the awareness, described by Equation (3). The blue arrows represent action of the brain upon the external world through the awareness, described by Equation (4). The green bar represents the external entropy production by the external world described by Equation (5). The internal world is quantitatively represented by brain size *N*. The external world represents tools and fire used in cooperation with other interacting human beings. The population size of a group *S*, which is the simplest quantity representing the external world, is used for present quantitative discussion. The external entropy production *P* is assumed to be proportional to *S* near the critical time, although this relation deviated from linearity very quickly.

In writing equations for this model, we use *N*; the effective size of the external world, *S*, which is represented by the population of a group to which the brain belongs; and the amount of the external entropy production *P*. A diagram of the growth model of the brain size and the birth of a cooperating group, which can produce external entropy production, is shown in Figure 2.

## 4. Birth of External Entropy Production by a Co-Development of Personal Brain and External Cooperative Group

### 4.1. Coupled Equations for Development of Personal Brain with External World

Although no mathematical analysis has existed so far [27,28] to the best knowledge of the authors, Figure 1 provides us as experimental data that shows qualitatively that brain size increases roughly from the time of using tools and fire. The present model was shown from (i) to (v) in the previous section. Although the dependence of the increasing speed of the brain size N(t) on the interacting group size S(t) is not known [23], it would be natural to assume that the brain growth rate is a function of personal brain size N(t) and population size S(t), to which the brain belongs. The diagram exhibiting the relation between the internal world represented numerically by brain size N(t) and the external world represented numerically by interacting group size S(t) through the awareness of the boundary world is shown in Figure 2.

The brain size N(t) increases by f(N,S), as well as the interaction of *N* with external group size *S*, and decays with a natural decay *D*,

(1) $$\frac{dN(t)}{dt}=f\left[N\left(t\right),S\left(t\right)\right]-D\left[N\left(t\right)\right].$$

This is the simplest model,

(2) f[N(t),S(t)]=CN(t)S(t),andD[N(t)]=γN(t),

where *C* is a coupling constant of *N* and *S*, and γ is a decay constant of *N*. Equation (1) will be

(3) $$\frac{dN(t)}{dt}=CN\left(t\right)S\left(t\right)-\gamma N\left(t\right).$$

The size of an external world *S* increases with the brain size *N*,

(4) $$\frac{dS(t)}{dt}=BN\left(t\right)-\mu S\left(t\right),$$

where μ is a decay constant of *S*. The nonlinear Equations (3) and (4) are a simplified coupled model of brain size *N* and the size of an external world *S*.

External entropy production per person *P* will be proportional in the beginning to the newly developed external world *S*,

(5) P(t)=AS(t),

where we set *A* to be a constant.

It is noted that the detailed mechanism of the birth of awareness at $N=N_{c}$, as well as the *N* dependence of the coupling constants A,B,C at $N>N_{c}$, are not discussed here and will be a subject of future study including brain science and evolutionary social science [29].

### 4.2. Linearized Solution with Numerical Values Obtained from the Historical Data

To solve *N* and *S* by linearizing from Equations (3) and (4) as

(6) $$N=N_{c}+n\left(t\right),\;\;\;S=S_{c}+s\left(t\right),$$

where $N_{c}=\gamma \mu /BC$ and $S_{c}=\gamma /C$ are the equilibrium stage of N−S space (i.e., dN/dt=dS/dt=0). The red circle in Figure 1 corresponds to $N_{c}\left(T_{c}\right)$. Inserting Equation (6) into Equations (3) and (4), we obtain linearized equations for *n* and *s* as

(7) $$\frac{dn(t)}{dt}=CN_{c}s\left(t\right),$$

(8) $$\frac{ds(t)}{dt}=Bn\left(t\right)-\mu s\left(t\right).$$

By setting n=n(0)exp(λt) and s=s(0)exp(λt), the eigenvalues λ are found to be

(9) $$\lambda =\frac{1}{2}\left(-\mu \pm \sqrt{\mu^{2}+4\gamma \mu }\right).$$

Considering that μ≫γ, the mode with the positive eigenvalue is

(10) λ≈γ.

The growth solution is consistent with the MEPP. The linear growth speed of the brain, coupled with the environmental growth at $N=N_{c}$, is determined by the decay speed of the brain. In order to obtain the information of numerical values utilized in this mode, we learn from Figure 1 as $N_{c}=480\;$cc and

(11) $$\gamma n\left(0\right)=\frac{dN}{dt}\left(N=N_{c}\right)∼200\;\mathrm{cc}/\mathrm{million}\;\mathrm{years}.$$

Assuming n(0)∼10cc as the fluctuation from $N_{c}$, the decay constant of the brain obtained by this model is γ∼20/million years, which is not very far from 5/million years, a known decay constant of RNA in born [30]. And assuming the critical group size $S_{c}∼10$ and the lifetime of a society 1/μ∼10 years, we estimate the coupling constants C∼2/million years and $B∼2\times 10^{3}/\mathrm{cc}\;\mathrm{million}\;\mathrm{years}$.

By the present analysis, the initiation of the external entropy production *P* with the formation of a society of multi-body life was also confirmed as a manifestation of MEPP. Although the constant *A* in Equation (5), which relates the amount of entropy production *P* and *S*, was not explicitly clarified in the present analysis, the growth rate is given by γ∼20/million years at $N=N_{c}$ and keeps increasing exponentially thereafter. It should be mentioned here that internal entropy production inside the brain also increases when $N>N_{c}$.

The coupling constant *C* in Equation (2) depends on the types of animals. The size of *C* for human beings is considered much larger than those of other animals due the accuracy of fingers and sensitivity of eyes obtained by the life style. $N_{c}$ and $S_{c}$ are inversely proportional to *C* as derived just below Equation (6). This suggests that the critical brain and society sizes for human beings may be smaller than those of other animals, and that this transition to external entropy production has so far been limited to human beings. Understanding the continued growth of the external entropy production is important, and for this purpose, future research on the detailed nonlinear mode will be necessary, in cooperation with research on cultural evolution theory [31].

## 5. Thermodynamic Evolution Theory of Life and Neo-Darwinian Theory

The late time evolution investigated in the present paper was shown to be consistent with the birth and early time evolution described in the preceding paper in the thermodynamic theory. The relationship between thermodynamic evolution theory and Neo-Darwinian theory is worth mentioning. We focus here on the targets and time scales of the two theories.

### 5.1. Neo-Darwinian Evolution Theory

Darwin defined the evolution of life as the survival of the fittest [32]. After Mendel’s discovery of the rule for inheritance over generation, Neo-Darwinism [33] appeared as a modified theory of evolution, taking into account the gene information and its mutation. Neo-Darwinian theory is qualitatively concerned with the diversity of species and with natural selection favoring organisms better adapted to the environment. The cause of changes is due to the mutation of genes, with possible epigenetic modification of DNA strands [34]. The former is known as neutral, and the latter is now being investigated to determine whether the effect is maintained over generations. Neo-Darwinian theory works with environmental change and the fitness of any part of the biological system at the time interval of interest. Evolutionary selection favors organisms that are better adapted to their environment. To predict change, information about environmental change is necessary.

### 5.2. Thermodynamic Evolution Theory

This theory has its basis in the second law of thermodynamics. This viewpoint insists that birth and successive evolution should consistently be described by the MEPP, when the local system under consideration is FFE and free for pumping of the produced entropy. The time scale is long because the thermodynamics are fundamentally stochastic. The target of MEPP is for a local system FFE in an infinitely large reservoir, and the time scale of MEPP is also long if the environment of the local system stays unchanged. The selection rule in evolution is limited to the amount of entropy production no matter what kind of change occurs in the environment.

### 5.3. Comparison of the Two Theories

Thermodynamic theory discusses entropy production only over a long time scale, while the fitness of the Neo-Darwinian theory is more restricted to a short time scale because fitness is a concept of a short time scale. The target of Neo-Darwinian theory covers any features of living bodies. The theory works well if fitness can be found at the time interval of interest.

Because the target is free, it may not be easy to discuss the fitness of the unknown future. On the other hand, the target quantity of the thermodynamic theory of evolution is only entropy production. The applicability is limited by the condition that the local system under consideration be open and far from equilibrium. The advantage of thermodynamic evolution theory is its ability to treat the birth and future of life consistently.

## 6. Human Being as a Symbiotic Existence of Multi-Cellular and Multi-Body Life

The important birth of awareness in the human brain was initially to avoid dangers from the environment, but after a while it was used for making tools, controlling fire, and initiating technology with a large amount of external entropy production, which is in accordance with the MEPP. The result of the present research implied that external entropy production is nothing but the birth of a second life, much like the birth of the first life, which started with a sudden increase in internal entropy production [1]. This situation reminds us of D.D., which switches between a single-cell state to a multi-cell state depending on the environmental condition as described in Section 2.2. As a result of evolution to external entropy production, the life of human beings has two aspects; multi-cellular life and multi-body life. The former has a long history of ∼2 billion years, and the latter social life has a relatively short history of ∼2 million years. This symbiotic structure has created some common features in the behavior of human beings.

### 6.1. Coexistence of Two Kinds of Entropy Production Mechanisms in a Body

Human society keeps increasing the amount of external entropy production, and at the same time individual human beings keep desiring individual survival. These two mechanisms function according to two different time scales and at two different locations in the body. Therefore, the behavior of human beings reflects these two functions. It is known that the consciousness of Homo sapiens reflects the function of the frontal cortex of the brain, because it is accompanied by awareness. The functions of other parts of the brain, as well as of the body, are carried out without consciousness and appear only during unconscious periods. This almost hidden factor may correspond to the phenomenon called instinct [35]. Therefore, thermodynamic evolution theory may contribute to research in the field of psychology, ethology and evolutional psychology.

### 6.2. Why Technology Grows Continuously?

Human beings keep promoting technology without knowing the goal. From a long-timescale consideration of external entropy production, human technology was fundamentally introduced by the thermodynamic principle, more precisely by the MEPP when the system is far from equilibrium (FFE). Therefore, the large amount of entropy production produced by modern technology is not necessarily a byproduct of convenience for human beings. On the contrary, the large amount of entropy production itself is, scientifically speaking, the goal of nature under FFE conditions, and tools and technology are only the means for external entropy production. This reversal of cause and result may help answer the question of why present human beings continue developing technology with increasing entropy production.

## 7. Conclusions

A new concept “external entropy production” proposed recently in thermodynamic evolution theory, in contrast to “internal entropy production” inside the living body, is investigated theoretically. It is pointed out from the archaeological data that growth of the human brain with the birth of awareness was indispensable for the invention of tools and control of fire, which triggered interaction with the external world. Brain size as the simplest measure of brain function and the interacting group size are useful metrics for a mathematical treatment for the birth of awareness and external entropy production. The birth of external entropy production is theoretically formalized by coupled equations of the personal brain size and the interacting population size through awareness. The theoretical results are compared with the data of the archaeological findings. The present analysis of the beginning of the external entropy production implies that external entropy production is nothing but a birth of the second life, as much as the birth of the first life, which started the sudden increase of internal entropy production. A long-timescale viewpoint of thermodynamic evolution suggests a new viewpoint on the meaning of technology and self-awareness, which have been interpreted only on short timescales of less than a few hundred years. This finding may have a new philosophical impact. It is suggested that the symbiotic existence of human beings with personal internal MEPP and external MEPP may be deeply related to the psychological phenomenon of instinct. It is hoped that a thermodynamic understanding of external entropy production may contribute to building countermeasures against global warming for future human life.

## Figures and Tables

**Table 1:** History of appearance time of ancient human beings and their brain sizes compared to a chimpanzee.

Time	Species	Brain Size
∼7 million years ago	Chimpanzee	387±57cc [19]
∼2.6 million years ago	*Australopithecus africanus*	458±6cc [20]
∼1.7 million years ago	Homo habilis	650±50cc [21]
∼1 million years ago	Homo erectus	950±143cc [22]
∼0.1 million years ago	Homo neanderthalensis	1565±101cc [23]

## Data Availability

The original contributions presented in this study are included in the article. Further inquiries can be directed to the corresponding author.
